# Alopecia and colon ulcers following azathioprine use in a patient with myasthenia gravis: A case report

**DOI:** 10.1097/MD.0000000000029986

**Published:** 2022-08-19

**Authors:** Wan-Yi Hsu, Pei-Chin Lin, Yi-Ching Liu, Lung-Chang Lin

**Affiliations:** a Division of Pediatric Emergency, Department of Pediatrics, Kaohsiung Medical University Hospital, Kaohsiung, Taiwan; b Graduate Institute of Medicine, College of Medicine, Kaohsiung Medical University, Kaohsiung, Taiwan; c Division of Hematology-Oncology, Department of Pediatrics, Kaohsiung Medical University Hospital, Kaohsiung, Taiwan; d Department of Pediatrics, School of Medicine, College of Medicine, Kaohsiung Medical University, Kaohsiung, Taiwan; e Division of Cardiology and Pulmonary, Department of Pediatrics, Kaohsiung Medical University Hospital, Kaohsiung, Taiwan; f Division of Neurology, Department of Pediatrics, Kaohsiung Medical University Hospital, Kaohsiung, Taiwan.

**Keywords:** alopecia, azathioprine, inflammatory bowel disease, Myasthenia gravis, NUDT15

## Abstract

**Rationale:**

Azathioprine is a purine analog (PA) used to treat myasthenia gravis (MG). However, some patients are sensitive to azathioprine and develop severe side effects, such as leukopenia, alopecia, and diarrhea soon after using the medication. Pharmacogenetics plays a crucial role in such intolerance.

**Patient concerns:**

A 16-year-old woman with MG developed hair loss, pancytopenia, bloody diarrhea, and fever shortly after azathioprine treatment.

**Diagnosis:**

Pharmacogenetic analysis revealed compound heterozygosity of the nudix hydrolase 15 (*NUDT15*) gene, which led to suppressed NUDT15 function. Colonoscopy revealed large ulcers with polypoid lesions in the terminal ileum, cecum, ascending colon, and rectum. These are the characteristics of inflammatory bowel disease (IBD).

**Interventions:**

Sanger sequencing of *NUDT15* gene and colonoscopy for bloody stool evaluation.

**Outcomes:**

The patient recovered completely from this acute episode after discontinuation of azathioprine treatment. Her hemogram turned back to normal range. There was also no blood in stool during follow-up.

**Lessons:**

Pharmacogenetic effects should be considered when prescribing PA medication. The possibility of secondary or concomitant autoimmune diseases must always be considered in patients with MG.

## 1. Introduction

Myasthenia gravis (MG) is a disorder of the postsynaptic receptors at the neuromuscular junctions of skeletal muscles. The first-line treatment for MG is pyridostigmine, an acetylcholinesterase inhibitor. However, if a patient’s symptoms do not improve after pyridostigmine treatment, an immune-modulating medicine, such as prednisolone or azathioprine, is often used as second-line treatment.^[1,2]^ Azathioprine (AZA) is a purine analog (PA) that blocks purine metabolism, inhibits DNA, RNA, and protein synthesis, and finally leads to cell death. Cells with high turnover rate are more susceptible to PA. Thus, the common adverse effects of PA include nausea, diarrhea, and cytopenia. AZA is widely used as an immunosuppressant for the treatment of hematological and autoimmune diseases. The usual dose of AZA for treating MG is 2 to 3 mg/kg/day, and can be increased gradually every 2 weeks. In addition to AZA and prednisolone, other immunomodulatory drugs, such as mycophenolate mofetil, cyclosporine, or cyclophosphamide, can also be used as second-line treatments.^[3]^ Pharmacogenetic studies have identified at least 2 important PA-metabolizing enzymes. They included thiopurine S-methyltransferase (TPMT) and nudix hydrolase 15 (NUDT15). The effects of these gene polymorphisms have been widely discussed in leukemia^[4–6]^ and inflammatory bowel disease (IBD)^[7–9]^; however, these effects have seldom been discussed in relation to neurological diseases.

We report a 16-year-old female MG patient with compound heterozygosity of *NUDT15* gene. The accumulation of toxic azathioprine intermediate metabolites due to suppressed NUDT15 activity causes severe adverse effects.

## 2. Case report

This 16-year-old female patient was diagnosed with MG and treated with pyridostigmine since December 2019. Despite pyridostigmine treatment, the patient still complained of diurnal ptosis and tiredness. Prednisolone was suggested but was strongly refused by the patient. Azathioprine treatment was initiated in January 2020. The dose was increased from 0.8 mg/kg/day to 1.6 mg/kg/day after 2 weeks. The patient began experiencing diffuse hair loss approximately 4 days after the dose increase. Besides, the patient complained of decreased appetite and fatigue. Azathioprine was discontinued after a 19-day treatment course. Seventeen days after discontinuation, the patient complained of fever and a sore throat. Frequent soft/watery stool passage and dull but worsening abdominal pain developed approximately 20 days after the discontinuation of azathioprine. The patient presented to our emergency room in February 2020 (27 days after azathioprine discontinuation) with the following vital signs: respiration of 20/min, blood pressure, 74/34 mm Hg; pulse rate, 89 beats/min; and body temperature, 35°C. Laboratory examination revealed pancytopenia, prolonged prothrombin time, and impaired renal function (Table 1). Abdominal computed tomography revealed ileocolitis and reactive lymphadenopathy in the right lower mesentery. The patient was admitted to the pediatric intensive care unit. Antibiotic treatment was initiated under the impression of impending septic shock and enterocolitis.

**Table 1:** Hemogram change before and after AZA treatment.

Admission	Day 1	Day 2	Day 5	Day 10	Day 28	
Days after AZA cessation	27	28	31	36	54	Before AZA started
WBC (10^3^/µL)	470	1000	3170	6490	7040	7980
ANC (%)	49%	59%	74%	75%	56.7%	68%
PLT (10^3^/µL)	4	35	78	199	223	361
Hb (g/dL)	7	7.8	7.5	10.2	11.4	12
Cre (mg/dL)	3.35				0.44	
PT (sec); PTT (sec)	24.0 (p′t)/10.9 (Crt.); 35.7 (p′t)/26.8 (Crt.)					
Transfusion		PLT 1U, pRBC, 2U				

ANC = absolute neutrophil count, Cre = creatinine, Crt. = control, Hb = hemoglobin, p’t = patient, PLT = Platelet, pRBC = packed red blood cells, PT; PTT = prothrombin time; partial thromboplastin time.

**Figure 1.**
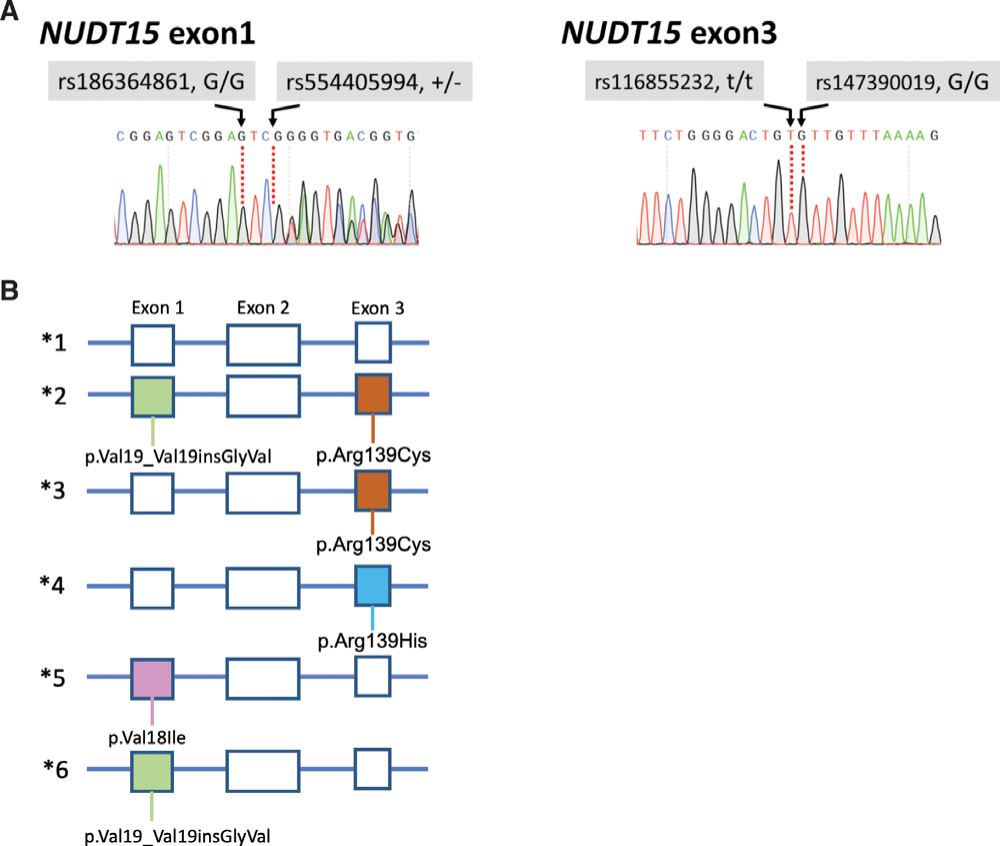
Results of *NUDT15* Sanger sequencing. (A) Sanger sequencing results of our patient. (B) Four relative common NUDT15 coding variants, result in 6 haplotypes.

Her white blood cell count rapidly rebounded after admission, without any intervention (Table 1). Tests for viruses (Epstein-Barr virus, adenovirus, cytomegalovirus, parvovirus B19, and HIV) and autoimmune antibodies (antinuclear antibodies, PR3-ANCA [proteinase 3 and antineutrophil cytoplasmic antibodies], anti-dsDNA antibodies, antimitochondrial antibodies, and anti–smooth muscle antibodies) produced negative results. The scheduled bone marrow examination was canceled because of the patient’s rapid recovery from pancytopenia. Genetic predisposition to azathioprine intolerance was suspected. Sanger sequencing of *NUDT15* gene revealed compound heterozygosity (diplotype *2/*3) (Fig. 1A,B; Table 2).

**Table 2:** NUDT15 SNP number and their nucleotide, amino acid change.

Reference SNP number	Nucleotide change	Amino acid change
rs186364861	c.G52A	p.Val18Ile
rs554405994	c.36_37insGGAGTC	p.Val19_Val19insGlyVal
rs116855232	c.C415T	p.Arg139Cys
rs147390019	c.G416A	p.Arg139His

The patient had persistent bloody stool and complained of lower abdominal pain after admission. Prominent bowel wall edema was observed on a sonographic examination conducted 10 days after admission. Colonoscopy with ascending colon biopsy was thus performed and revealed large ulcers with polypoid lesions in the terminal ileum, cecum, ascending colon, and rectum. Pathological examination revealed necrotizing granulomas without caseation or inclusions. Pathogens (Salmonella, Shigella, Campylobacter, *E. coli* O157, Vibrio, *Clostridium difficiles*, ameba, and parasite ova) were not detected in the patient’s stool. The final diagnosis was typhlitis.

After our patient was discharged from the hospital, she had no occult blood in the stool examination and no iron-deficiency anemia noted during the outpatient follow-up. Her hair amount returned to her previous level within 3 months after AZA discontinuation. The patient still had symptoms of diurnal ptosis and blurred vision under pyridostigmine treatment alone. We started prednisolone treatment (45 mg/day then tapered down gradually), and then switched to cyclosporine after 2 months because of poor symptom control. Ptosis improved with cyclosporine (1.1 mg/kg/dose, twice per day).

## 3. Discussion

**Figure 2.**
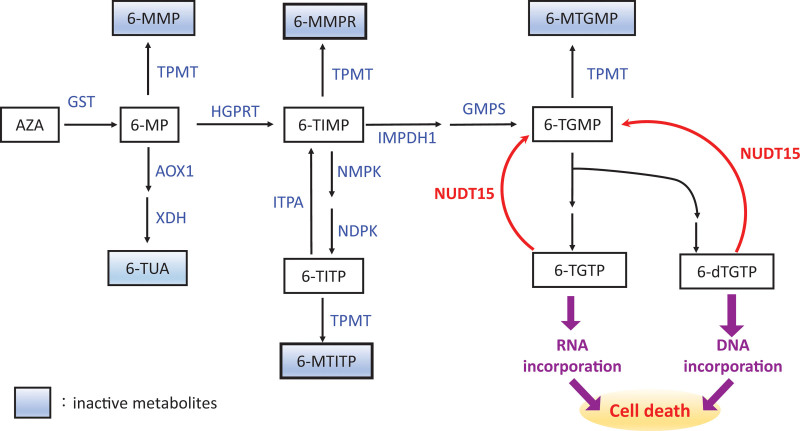
Pathway of azathioprine metabolism. 6-dTGTP = 6-deoxy-thioguanine triphosphate, 6-MMP = 6-methylmercaptopurine, 6-MMPR = 6-methylmercaptopurine ribonucleotide, 6-MTGMP = methyl 6-thioguanine monophosphate, 6-MTITP = methyl 6-thionosine triphosphate, 6-TGMP = 6-thioguanine monophosphate, 6-TGTP = 6-thioguanine triphosphate, 6-TIMP = 6-Thionosine monophosphate, 6-TITP = 6-thionosine triphosphate, AOX1 = aldehyde oxidase type 1, GMPS = guanosine monophosphate synthetase, GST = glutathione s-transferases, HGPRT = hypoxanthine-guanine phosphoribosyltransferase, IMPDH1 = inosine 5-monophosphate dehydrogenase type 1, ITPA = inosine triphosphate pyrophosphatase, NDPK = nucleoside diphosphate kinase, NMPK = nucleoside monophosphate kinase, TPMT = thiopurine *S*-methyltransferase, TUA = thiouric acid, XDH = xanthine dehydrogenase/oxidase.

Azathioprine (AZA) is a prodrug. It is converted to the active cytotoxic metabolites 6-thioguanine triphosphate (6-TGTP) and 6-deoxy-thioguanine triphosphate (6-dTGTP) by various enzymes (Fig. 2). The diminished activity of TPMT and NUDT15 causes overaccumulation of 6-TGTPs and 6-dTGTPs and, thus strongly augments drug efficacy and exacerbates the severity of adverse effects. Genetic polymorphisms could affect enzyme activity. TPMP is the most widely discussed 1. At least 45 *TPMP* variants have been reported, 10 of which cause considerable clinical toxicity. Studies have reported that the prevalence of *TPMP* polymorphisms is 10% to 15% among Caucasian and African populations, but much lower among Asian populations.^[10,11]^

The association of PA intolerance with *NUDT15* variants was first reported in a patient with IBD in 2014.^[11]^ To date, at least 20 of 4,430 known single-nucleotide variants significantly reduce NUDT15 activity.^[11,12]^ In contrast to the high prevalence of *TPMT* polymorphism in western countries, the normal *NUDT15* diplotype occurs in 99%, 77.4%, and 86.4% of Europeans, East Asians, and South Asians, respectively.^[11,13]^ Among the *NUDT15* diplotypes, p.Arg139Cys (rs116855232, haplotype *3) most strongly inhibits NUDT15 activity and exhibits high linkage disequilibrium with p.Val19_Val19insGlyVal (rs554405994 coexisting with rs116855232 is called haplotype *2) (Fig. 1B; Table 2).^[13]^ Our patient exhibited compound heterozygosity with haplotypes *2 and *3. Pediatric leukemia studies have revealed that patients with this variant can tolerate only 8% of the usual PA dose and account for approximately 22% of PA intolerance cases.^[14,15]^ In vivo and in vitro studies of NUDT15*3 have revealed that this variant does not affect enzyme activity; instead, it causes instability in NUDT15 protein structure, leading to early protein degradation in cells. Physiologically, diplotype *2/*3 makes enzyme NUDT15 no function at all.^[16]^

Administering full doses of PA medication to patients with susceptible *NUDT15* and TPMT variants can cause alopecia, leukopenia, and gastrointestinal discomfort.^[10,14,17,18]^ Like our patient, these symptoms develop rapidly, often within 2 weeks of medication initiation. As *NUDT15* polymorphisms are prevalent among Asian populations, checking for NUDT15 variants before administering PA to Asian patients is highly recommended. Sanger sequencing may be the most cost-effective method for detecting these variants.^[19,20]^

In addition to the *NUDT15* variant, the colonoscopy findings in our patient are worth discussing. Ulcers in the terminal ileum and cecum are common features of IBD. Approximately 15% of patients with MG have been reported to have a second autoimmune disease, but IBD has rarely been reported among these patients.^[21]^ According to previous studies,^[22–24]^ IBD is often been diagnosed long before MG development; however, 1 patient with MG diagnosed before IBD was reported.^[25]^ Our patient denied frequent abdominal pain, loose stools, or tenesmus during the outpatient follow-up. She has no occult blood in the stool examination and no iron-deficiency anemia. However, her colonoscopic examination results were still noteworthy, and her gastrointestinal condition should be closely monitored in the future.

In conclusion, the pharmacogenetic effects of genetic variants should be considered before a patient is prescribed a medication. In the decision to initiate PA treatment, especially in Asian patients, *NUDT15* variants should be considered. Because DNA sequencing techniques are not universally accessible, starting with a low dose of PA, closely monitoring hemogram trend and symptoms of adverse effects are crucial.

## Author contributions

Data collection: Wan-Yi Hsu, Yi-Ching Liu.

Literature reviews and curation: Pei-Chin Lin, Lung-Chang Lin.

Patient care: Wan-Yi Hsu, Pei-Chin Lin, Yi-Ching Liu, Lung-Chang Lin.

Writing – original draft: Wan-Yi Hsu.

Writing – review & editing: Pei-Chin Lin, Yi-Ching Liu, Lung-Chang Lin.
